# Human Metabolic Enzymes Deficiency: A Genetic Mutation Based Approach

**DOI:** 10.1155/2016/9828672

**Published:** 2016-03-09

**Authors:** Swati Chaturvedi, Ashok K. Singh, Amit K. Keshari, Siddhartha Maity, Srimanta Sarkar, Sudipta Saha

**Affiliations:** ^1^Department of Pharmaceutical Sciences, Babasaheb Bhimrao Ambedkar University, Raebareli Road, Vidyavihar, Lucknow 226025, India; ^2^Department of Pharmaceutical Technology, Jadavpur University, Kolkata 700032, India; ^3^Dr. Reddy's Laboratories Limited, Bachupally, Hyderabad, Telangana 502325, India

## Abstract

One of the extreme challenges in biology is to ameliorate the understanding of the mechanisms which emphasize metabolic enzyme deficiency (MED) and how these pretend to have influence on human health. However, it has been manifested that MED could be either inherited as inborn error of metabolism (IEM) or acquired, which carries a high risk of interrupted biochemical reactions. Enzyme deficiency results in accumulation of toxic compounds that may disrupt normal organ functions and cause failure in producing crucial biological compounds and other intermediates. The MED related disorders cover widespread clinical presentations and can involve almost any organ system. To sum up the causal factors of almost all the MED-associated disorders, we decided to embark on a less traveled but nonetheless relevant direction, by focusing our attention on associated gene family products, regulation of their expression, genetic mutation, and mutation types. In addition, the review also outlines the clinical presentations as well as diagnostic and therapeutic approaches.

## 1. Introduction

Understanding of metabolism and energy flow through cells has recently gained considerable interest [1]. Inborn error of metabolism (IEM) is a group of disorders characterized by a single gene defect, which blocks some vital steps in the normal metabolic pathway ensuing in deposition of substrate or insufficiency of the product for normal organ functions [2]. Diagnosis is of foremost choice not only for treatment and prognosis but also for genetic counseling [3]. Enzyme deficiency is thought to be genetically inherited almost always in a recessive fashion, as it is mainly the result of “loss-of-function” mutations [4]. This can be inherited either as autosomal recessive (both of the parents do not have disorder but each of them carries faulty gene and delivers it to the child) or as X-linked recessive (only the mother carries the affected gene on the X chromosome and conveys it to the child) [5].

The inheritance of the majority of metabolic disorders is rare [6] and the age of onset is extremely variable; however, IEMs afflict mostly the paediatric population [2]. Early detection of IEM correlates with significant reduction in associated disabilities and deaths [6]. Genetic mutation is also responsible for enzyme defect that regulates enzyme protein interaction during transportation and binding of cofactors. As a result, there is a modification in cellular chemistry either by diminution of essential component or by accumulation of toxic substances [4].

Treatment approaches for metabolic disorders are based on symptomatic therapy which may include (a) modification of metabolism process through restriction of attachment of precursor with enzymes; [4] (b) provocation and stabilization of residual enzyme activity using cofactors or vitamins; (c) blocking the production of toxic metabolites using detoxifying agents; [7] (d) replacement therapy to supply exogenous functional enzymes; (e) endogenous production of enzymes through organ transplantation; (f) gene therapy to replace defective gene [4]; (g) avoidance of catabolism at all stages of treatment. Nonetheless, the nutrition therapy is considered as an integral part for the treatment of IEM. Few parameters are essential for assessment of IEM which include nutrient intake, growth rate, and laboratory values monitoring [7, 8].

**Figure 1:**
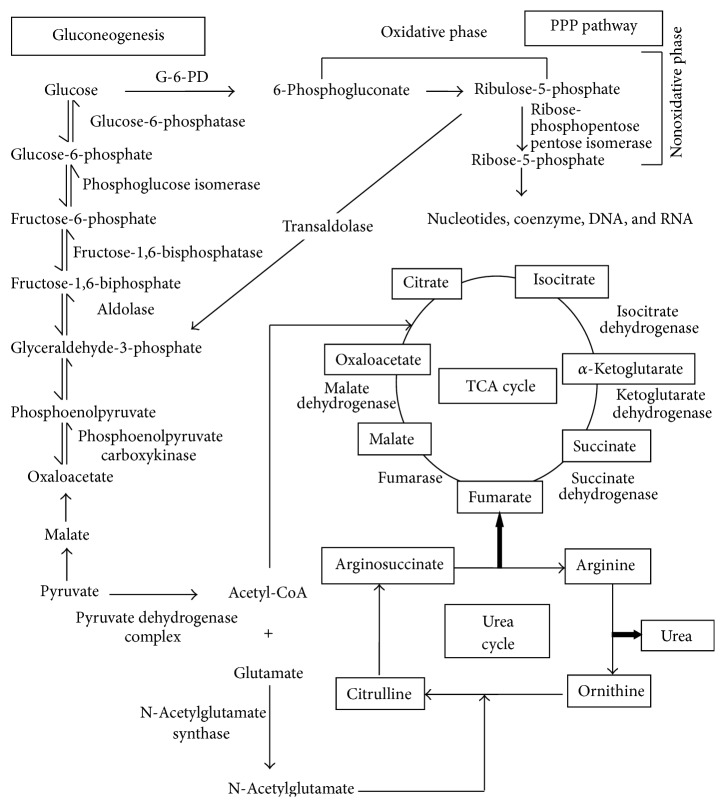
Interlinking between various metabolic pathways (gluconeogenesis, Krebs cycle, urea cycle, and pentose phosphate) and various enzymes responsible for metabolism.

Hitherto, despite the appearance of quite a few excellent reviews in the field of IEM of the literature, no review has focussed on summarizing the real correlation of human metabolic enzyme deficiency (MED) with inborn error of metabolism (IEM), particularly in the sense of genetic mutation. The aim of this review is, therefore, to provide the most advanced information about the key enzymes critically involved in diverse well-known metabolic pathways like gluconeogenesis, Krebs cycle, urea cycle, and pentose phosphate (PPP) pathway (Figure 1). The emphasis here is given to how genetic mutation or altered gene expression affects MED-associated disorders.  Table 1 represents the summarized form of metabolic enzymes deficiency disorders and genetic mutations.

## 2. Metabolic Enzymes Deficiency: Cause and Complications

### 2.1. Glucose-6-phosphatase (G6Pase) Deficiency

G6Pase helps in the formation of glucose-6-phosphate from glucose in the lumen of endoplasmic reticulum (ER) [9, 10]. Herein, the enzyme is a part of the multicomponent system, including several integral membrane proteins, G6Pase catalytic subunit (G6PC), a regulatory Ca^2+^ binding protein, and glucose-6-phosphatase translocase (G6PT) [11]. G6Pase activity is restricted to the various gluconeogenic tissues like liver [12], kidney [13], small intestine [14], and *β*-cells of the endocrine pancreas [9].

G6Pase enzyme is encoded by G6PC1, G6PC2, and G6PC3 genes which are responsible for metabolic disorders. G6PC1 is expressed in the liver, kidney, and small intestine, whereas G6PC2 is expressed in the pancreas and G6PC3 is expressed ubiquitously in the human body [15, 16]. G6PC1 and G6PC3 are located on the 17q21 chromosome and G6PC2 is on the 2q31 chromosome. The cytosolic glucose-6-phosphate is transported to ER through SLC37A4 encoded gene [17, 18]. Deficiency of G6Pase activity in liver, kidney, and intestinal mucosa with excessive accumulation of glycogen in these organs leads to glycogen storage disease (GSD) type 1 (Von Gierke's disease). The latter is considered as acute metabolic disorder preferably characterized by hypoglycemia. There are two main types of glycogen storage diseases: the first is due to a defect in G6PC, called GSD type 1a, and the second one is due to the defect in G6PT, called GSD type 1b [19, 20].

GSD-1a patients are clinically diagnosed with prompt induced hypoglycemia and hyperlactacidemia in the neonatal period. Protruded abdomen due to pronounced hepatomegaly is the first symptom developed around 3 months of age. Moreover, the other biological hallmarks are hyperlipidemia, hyperuricaemia, round doll-like face, developmental delay, and late onset of puberty [20–22]. The clinical signs are chronic acidosis and hypertriglyceridemia which led to the development of osteopenia and enlarged kidneys. Long term complications may be the hepatocellular adenomas, renal complications, hyperuricaemia, and severe hypertriglyceridemia which may cause risk of pancreatitis and pulmonary hypertension [23]. In GSD-1b patients along with these symptoms, patients are also diagnosed with neutropenia, which is responsible for development of Crohn's disease [21, 24]. In the recent studies, the antibacterial flagellin antibodies (anti-CBir1) detection in GSD-1b patients is another indication of Crohn's disease and this antibody level increased during disease state. In GSD-1b patients, splenomegaly is more common along with hepatomegaly, which is rarely found in GSD-1a patients [20, 25].

Previously, liver biopsy was the main diagnosis for the detection of G6Pase disorder. Recent advances in molecular biology involve DNA based diagnostic tests and genes cloning and G6Pase mutation database helps in diagnosis. Hitherto, more than 80 separate mutated genes are identified for G6Pase gene family, which are directly or indirectly responsible for G6Pase activity. These include missense (E110Q, D38V, P178S, W236R, R295C, and L345R), nonsense (Q347X and R170X), insertion/deletion (822delC and 813insG), and codon deletion (DF327) mutations, which are capable of reducing the G6Pase activity [19].

Naturally occurring dog and transgenic mice models are used for the study of GSD-1a whereas transgenic mice models are for GSD-1b [10, 26]. The transgenic mouse model shows all the symptoms of human GSD-1a, that is, hypoglycemia, hepatomegaly, nephromegaly, growth retardation, hyperlipidemia, mild lactic acidemia, and hyperuricemia [10, 26]. Crossbreeding of Beagle and Maltese (with Met121Ile mutation) showed mutation of G6PC gene with symptoms of GSD-1a [26, 27]. These animal models would serve as a useful tool for the understanding of the pathophysiological conditions and therapeutic approaches of GSD-1a deficiency [26]. Gene therapy related to adenoviral and adeno-associated virus vectors is another important therapeutic approach for G6Pase-*α* [28, 29]. Moreover, measurement of granulocyte colony-stimulating factor (GCSF) is an important parameter for GSD-1b diagnosis, as G-CSF may increase the number and improve the function of circulating neutrophils, and G-CSF may improve the symptoms of Crohn-like inflammatory bowel disease in individuals with GSD-1b [30, 31].

Corn starch and other carbohydrates are the primary treatment for G6Pase deficiency [32]. It is also necessary to normalize other physiological parameters during disease state of G6Pase deficiency. Allopurinol and angiotensin-converting enzyme (ACE) inhibitors are used as supplementary drug to lower the uric acid and microalbuminuria [20]. Adjunct therapy during G6Pase deficiency includes lipid lowering drugs and potassium citrate [26, 33, 34]. Liver transplantation in the patient with GSD-1a can be performed if dietary therapy becomes unresponsive to hepatocellular adenoma and tumors. Bone marrow transplantation can be undertaken for the patients with GSD-1b related myeloid deficiencies [26, 34–39].

### 2.2. Fructose-1,6-bisphosphatase (FBPase) Deficiency

FBPase is an unique enzyme in the gluconeogenetic pathway, regulated via alteration of the active (R) and inactive (T) conformational isomeric states [11, 40], which catalyzes the magnesium dependent reversible production of fructose-1,6-bisphosphate from fructose-6-phosphate and inorganic phosphate [41]. The molecular weight of human FBPase is 36.7 KDa and consists of four identical subunits of one substrate and one allosteric site. FBPase activity is regulated by fructose-2,6-bisphosphate (binds to substrate site) and adenosine monophosphate (binds to allosteric site). This enzyme is encoded by the FBP1 gene in liver and kidney at 9q22.2 and q22.3 chromosomal site [42].

FBPase deficiency is a metabolic recessive disorder in the liver that is characterized by the life-threatening episodes of hyperventilation, hypoglycemia, apnoea, lactic acidosis, and ketosis [43, 44]. Kikawa, for the first time, identified the mutations of FBPase in ten patients of eight unrelated families, suggesting that FBP1 gene mutation is responsible for FBPase deficiency. To date, several different mutations have been published on individuals with FBPase deficiency. Among them, 960/961insG, G164S, A177D, and E30X were reported in Japanese unrelated families. Recently, two new FBP1 gene mutations, F194S and P284R, were identified in a Japanese female patient with FBPase deficiency. p.G260R, c.778G>A, and p.Y216X are the newly identified mutations in Swedish patients [43, 45]. Generally, the mutations are widespread throughout the FBP1 gene, and each mutation was found in one case or family, with an exception: an insertion of guanine at position 960 that has been found in several patients with different ethnic backgrounds [46]. FBP1 gene was downregulated in Ras-mediated transformation and in gastric carcinogenesis and NF-kappa-B is involved in initiation of FBP1 gene downregulation (Warburg effect) [45–49].

The diagnosis of FBPase enzyme deficiency was determined through spectrophotometric and load tests (radiochemical) [50, 51] in liver, kidney [52], and jejunum [32]. Calcitriol stimulated FBP1 gene expression is similar to expression of vitamin D receptor [53]. The measurement of FBPase deficiency is observed in leucocytes [54, 55]. Later, similar activity is determined in monocytes where stimulation with calcitriol resulted in four- to sixfold enhancement of activity. Further immunoblotting technique revealed the presence of enzymes in monocytes but not in lymphocytes [56]. Moreover, both clinical symptoms and mutation analysis are the common methods for FBPase activity. In addition, activity assessment of liver tissue is generally used for a reliable diagnosis [49].

Glucose (10–12 mg/kg/minute, newborns) and bicarbonate (200 mmol/24 h) are given to control hypoglycemia and acidosis. Starch and gastric drip are frequently given during treatment but not sucrose, sorbitol, fructose, fat (20–25%), and protein (10%) [57].

Enhancement of FBPase activity during type 2 diabetes is the primary role as this enzyme promotes gluconeogenesis [58]. However, antidiabetics do not reduce gluconeogenesis, and therefore inhibition of FBPase activity is required separately [58–60]. The uses of selective FBPase inhibitors (adenosine monophosphate) and structure guided design strategy are the important parameters for FBPase activity. In light of the same, quite a few FBPase inhibitors are in their different stages of ongoing clinical trials (CS-917 and MB07803) [58].

### 2.3. Phosphoenolpyruvate Carboxykinase (PEPCK) Deficiency

PEPCK, an essential marker for gluconeogenesis, catalyzes the conversion of phosphoenolpyruvate to oxaloacetate. There are different isoforms of PEPCK, that is, PEPCK1 (cytosolic) and PEPCK2 (mitochondrial) [61]. PEPCK1 is localized on chromosome 20q13.31 and encodes a 622-amino acid polypeptide with 91% sequence similarity to that of the rat, whereas PEPCK2 is localized on chromosome 14q11.2 and encodes a 640-amino acid polypeptide with 78% sequence identity to that of the human PEPCK1 [61, 62]. PEPCK1 is regulated by the mitochondrial GTP-dependent pathways, including hormones, substrate supply, and purine nucleotides.

Although this enzyme helps in gluconeogenesis, it has an important role in glyceroneogenesis where it helps in the synthesis of glyceride-glycerol from glucose or glycerol in adipose tissue and liver [63, 64]. It plays another role in citric acid cycle and helps in the entry of carbon skeletons to amino acids [65]. Recently, it has been reported that the role of this enzyme in mammary gland epithelial cells (HC11 cells) is derived from COMMA-D epithelial cells and isolated from the mammary gland of pregnant BALB/c mice [66, 67]. Apart from this, PEPCK2 is known for its ability to fix carbon dioxide by converting pyruvate into oxaloacetic acid (Wood-Werkman pathway) [68]. Moreover, PEPCK2 is principally involved in gluconeogenesis, providing the cytosolic NADH through its conversion to pyruvate from lactic acid. This enzyme deficiency is an autosomal recessive disorder whose phenotype is not expressed clearly. Lactic acidosis and hypoglycemia are the primary symptoms for PEPCK deficiency. Reye syndrome develops due to inhibition of gluconeogenesis which, in turn, is due to PEPCK enzyme deficiency [69].

The specific symptoms of PEPCK deficiency are associated with lactic acidosis, hypoglycaemia, hepatomegaly, glucagon insensitivity, failure to thrive, Fanconi syndrome, developmental delay, hypotonia, and massive fat deposition in liver and kidneys [70].

Treatment of PEPCK deficiency includes the maintenance therapy similar to FBPase deficiency to treat acute attacks (glucose and bicarbonate infusions). There is no specific treatment other than maintaining normoglycaemia and correcting metabolic disorders.

### 2.4. Pyruvate Dehydrogenase Complex (PDHC) Deficiency

PDHC is critically involved in the conversion of pyruvate to acetyl-coenzyme A. The complex is composed of three different enzymes which are pyruvate decarboxylase (E1), dihydrolipoyl transacetylase (E2), and dihydrolipoyl dehydrogenase (E3). This complex requires five coenzymes for the reaction, three prosthetic groups (thiamine pyrophosphate, FAD, and lipoic acid), and two other carriers (coenzyme A and NAD) [71]. PDHC deficiency is considered one of the most common genetic as well as neurodegenerative disorders generally associated with abnormal mitochondrial metabolism. It is an extremely heterogeneous condition, also one of the X-linked diseases in which heterozygous female exhibits severe symptoms [72–75].

Around 200 cases of PDHC deficiency were reported previously where mutation at E1*α* subunit of Xp22 chromosome has occurred. 80 different mutated genes from E1*α* subunit had been identified which are responsible for deficiency [76–78]. Few cases of deficiency were not clearly understood and it is assumed that this happened due to alteration of recessive genes (1 : 50000 cases in males) [79].

Clinical spectrum of PDHC deficiency is broad and is divided into neurological as well as metabolic manifestations. Neurological presentation includes hypotonia, spasticity, dysplasia of the dentate nuclei, pachygyria, mental retardation, and Leigh syndrome. The metabolic manifestation of this enzyme deficiency occurs at neonatal period due to lactic acidosis. Maple syrup urine disease (MSUD) and energy metabolism disorder occurred during PDHC deficiency due to increased levels of plasma pyruvate, lactate, and *α*-ketoglutarate [80]. Sometimes, neonatal lactic acidosis along with respiratory disturbances was observed during this enzyme deficiency state [81]. Mutations in pyruvate dehydrogenase phosphatase gene have also been recently identified [79, 82].

Diagnosis is based upon the laboratory measurements of lactate and pyruvate in blood and cerebrospinal fluid (CSF). High blood lactate and pyruvate levels in blood and cerebrospinal fluid with or without lactic acidemia suggest the deficiency of PDHC. Furthermore, lactate-to-pyruvate ratio is diagnostically useful to differentiate PDHC deficiency from other forms of congenital lactic acidosis at higher lactate levels (>5 mmol/L). A low L : P ratio is observed in inherited disorders of PDHC deficiency. As in the case of PDHC deficiency, the mutation arises from the germ cells of one of the parents and the majority of children die before they reach their adulthood; the prenatal diagnosis is extremely useful for diagnosing patients before they are born so that treatment can be initiated immediately after birth. The identification of mutated gene deficit and genetic analysis in pregnancies is one of the most reliable methods for prenatal diagnosis [82]. In prenatal diagnosis, cultured chorionic villus cells are the most reliable to measure enzymatic activity. In male foetus, it is easy to diagnose with confidence, but in female foetus, it becomes difficult due to extreme skewing of X-chromosome inactivation.

Treatment includes the ketogenic diet, an important rational strategy for PDHC deficiency, but it does not improve the neurological symptoms and structural damage in the brain. Thiamine at variable doses and dichloroacetate at 50 mg/kg were found to be effective in some patients for potential treatment of PDHC deficiency and around 40 cases have been treated with the same. The combination of DCA and thiamine can be given in chronic cases but thiamine with ketogenic diet should be tried in each and every patient [72, 82].

### 2.5. Succinate Dehydrogenase (SDH) Deficiency

SDH (succinate ubiquinone oxidoreductase) is composed of heterotetrameric protein with SDHA and SDHB subunits, which bulge into mitochondria and coupled with inner membrane by SDHC and SDHD subunits (ubiquinone attachment site). All these subunits together are called complex II, which helps in Krebs cycle [83]. The four subunits of SDH are encoded by four nuclear genes located on chromosomes 1p35-p36.1, 5p15, 1q21, and 11q23 [84, 85]. Leigh's syndrome, also known as subacute necrotising encephalomyelopathy (SNEM), is a neurodegenerative disorder and is associated with SDH deficiency due to mutation [86].

PGL4 syndrome (pheochromocytoma/paraganglioma syndrome type 4) is characterized by gastrointestinal stromal tumors and renal tumors and are usually classified as carcinoma. PGL4 syndrome is caused by SDHB deficiency which is due to the missense mutation. Moreover, hereditary paraganglioma and pheochromocytoma is the main disease state for SDH deficiency which occurs due to mutation of SDHB, SDHC or SDHD subunits [87, 88]. The similar diseases also occur due to mutation of SDHA and SDH subunits assembly factor 2 [89, 90]. Various disorders such as Leigh syndrome, progressive myopathy, ophthalmoplegia, optic atrophy, and ataxia are the main clinical manifestations during SDH deficiency [91, 92]. Treatment of this enzyme deficiency is symptomatic.

### 2.6. Fumarase or Fumarate Hydratase (FH) Deficiency

FH catalyzes the conversion of fumarate to malate which is responsible for autosomal recessive disorder in the Krebs cycle. There are two types of fumarase isoenzymes, present in cytosol and mitochondria. Mitochondrial fumarate hydratase is responsible for catalytic reversible conversion of fumarate to malate during citric acid cycle whereas cytosolic fumarase is involved in fumarate metabolism during urea cycle [93].

The mutant alleles of the FH gene are located on human chromosome 1 at position 1q42.1. [94–96]. However, genetic analysis revealed that mutation occurs at 435insK chromosome for several patients (GenBank U59309) whereas the other mutations seemed to be private mutations [97–100]. The FH gene is similar to tumor suppressor gene, related to renal cell cancer and hereditary leiomyomatosis [101, 102].

Fumaric aciduria occurs during FH deficiency, characterized by neurological impairment, encephalopathy, and seizures, which causes death in childhood [86]. Neuropathological changes include choroid plexus cysts, polymicrogyria, and hypomyelination which occurs at white matter of brain in old ages [97, 103]. FH enzyme concentration is measured in blood leukocytes, liver, and skin fibroblasts during deficiency state (via coupling reaction with malate dehydrogenase) [86, 101]. Unfortunately, to date, there is no specific treatment yet to be employed effectively.

### 2.7. Glucose-6-phosphate Dehydrogenase (G6PD) Deficiency

G6PD works in pentose phosphate (PPP) pathway and helps in the reduction of nicotinamide adenine dinucleotide phosphate (NADPH). G6PD serves as antioxidant enzyme where it donates one electron to oxidised glutathione (GSSG) which converts into reduced glutathione (GSH) [80, 104]. The deficiency syndrome also relates to X-linked hereditary disorder [104–107]. G6PD deficiency occurs everywhere due to* de novo* mutations [108]. There are 160 different mutated genes, which is responsible for G6PD deficiency. The gene involved in the disease is located on Xq28, containing 13 exons, and encoded by a protein with 515 amino acids. A G6PD gene mutation distribution rate differs from one geographic area to another [104, 109]. G6PD A-202(G→A)/376(A→G) is the most widespread mutation in the African continent. A G6PD gene mutation called “Mediterranean” has also frequently been distributed from Mediterranean and Middle Eastern countries to the Indian subcontinent. It is the most common mutation among patients from the northern provinces of Iran. Apart from these, Chatham and Cosenza, the two other common G6PD gene mutations, have the highest frequency rates in those areas [110–112].

The patients with G6PD deficiency suffer from cyanosis, headache, fatigue, tachycardia, dyspnoea, lethargy, lumbar/substernal pain, abdominal pain, splenomegaly, hemoglobinuria, and/or scleral icterus. Moreover, the broken down products of hemoglobin may accumulate in the blood, causing jaundice, and are excreted in urine, causing dark brown discoloration [80].

### 2.8. Ribose-5-phosphate Isomerase (RPI) Deficiency

RPI is an enzyme of PPP pathway, which catalyzes the conversion between ribulose-5-phosphate (Ru5P) and ribose-5-phosphate (R5P). With a much lower number of diagnosed patients, RPI deficiency is currently the rarest disease in the world [113, 114]. During RPI deficiency, the human is attacked by epilepsy, followed by weakening of speech, vision, hand coordination, and walking [114].

It contains 2p11.2 gene having 9 exons and 311 amino acids. Deficiency of this enzyme is found to be caused by a combination of two mutations. The first is a deletion (c.540delG) and the second is a missense mutation (c.182C>T) of 2p11.2 gene [113].

The disease is clinically specified by leukoencephalopathy and mild peripheral polyneuropathy. Other neurological parameters like prominent cerebellar ataxia, nystagmus, bilateral optic atrophy, and spasticity were also observed during this enzyme deficiency [113].

The levels of D-xylulose, ribose, ribitol, and arabitol are increased in urine during deficiency state and therefore the diagnosis of this enzyme deficiency is performed by the concentration of sugars and polyols in the urine sample. Diagnosis may also be undertaken through the enzymatic assay in fibroblasts and sequence analysis of R5P gene. There is no specific treatment available for such deficiency [80].

### 2.9. Transaldolase (TALDO) Deficiency

TALDO is a nonoxidative enzyme of pentose phosphate pathway, which is involved in making a correlation between PPP and glycolysis pathways. Transaldolase (TALDO) deficiency is a newly recognized metabolic disease, which has been reported so far in 2 patients presenting with liver failure and cirrhosis. Deficiency of this enzyme shows elevation of polyols and seven-carbon sugars (erythritol, arabitol, and ribitol) in the body [113].

TALDO gene is composed of 11p15.5-p15.4 chromosome with another pseudogene at 1p34.1-p33 chromosome. TALDO deficiency is caused by mutation in TALDO 1 gene in the form of c.575C>T (p.Arg192Cys), c.574G>A (p.Arg192His), and c.512-514delCCT [115, 116].

The common clinical symptoms include bleeding problems, hepatosplenomegaly, enlarged clitoris, liver cirrhosis, thrombocytopenia, elevated bile acid with normal bilirubin, and mildly prolonged prothrombin time during deficiency of TALDO. In addition, the patient may suffer from respiratory failure, progressive myocardial hypertrophy, bradycardia, severe lactic acidosis, and liver failure [117–119].

The deficiency of TALDO enzyme is diagnosed by elevated concentrations of ribitol, arabitol, and erythritol in urine sample. The elevated concentrations of these markers are more prominent in neonatal stage and older patients [118, 119]. There is no specific treatment available for TALDO deficiency. Liver transplantation is an alternative approach for liver cirrhosis which occurred during this enzyme deficiency [117, 118].

### 2.10. N-Acetylglutamate Synthase (NAGS) Deficiency

NAGS is present in the small intestine and liver which acts as an important enzyme to regulate ureagenesis [120–122]. In urea cycle, N-acetylglutamate (NAG) is required as the allosteric activator of carbamylphosphate synthetase, a rate limiting enzyme of the urea cycle. NAGS catalyzes the conversion of glutamate to NAG by combining with Acetyl-CoA. This is why the deficiency in NAGS leads to hyperammonemia [123].

NAGS deficiency is the rarest autosomal, recessive, inherited metabolic disorder, which is characterized by hyperammonemia [120, 121, 124, 125]. NAGS deficiency is clinically characterized by seizures, poor feeding, hyperammonemia, coma, and chronic headaches [123, 124]. The biochemical estimation of all intermediates except elevated plasma ammonia and glutamine shows normal results. Moreover, urinary orotic acid level is not elevated during deficiency of this enzyme [126, 127]. However, diagnosis can be achieved by hepatic enzymatic studies [127] but in some cases it is not reliable [128, 129]. Therefore, accurate diagnosis is performed by cloning of the NAGS gene [127]. The gene is located on chromosome 17q21.31 consisting of 7 exons and 6 introns. Mutations in the NAGS gene include 15 missense, 1 nonsense, 4 frame-shift, and 2 splice-site mutations [130].

NAGS deficiency is the only inherited urea cycle disorder that can be specifically and effectively treated by a drug N-carbamylglutamate (NCG) which appears to be beneficial for the treatment of hyperammonemic conditions and increases the rate of ureagenesis [131].

During deficiency of this enzyme, arginine supplement, sodium phenylacetate, sodium benzoate, and sodium phenylbutyrate are generally given to scavenge the excess ammonia [132].

## 3. Conclusion

Contribution of mutational approach to detection of real cause associated with metabolic enzymes deficiency has allowed the design of “tailor-made” therapeutic strategies to alleviate most of the metabolic diseases. The original cause of most metabolic enzyme disorders is an IEM, particularly gene mutations. However, there is a significant level of tolerance in the system. For example, a mutation in one enzyme does not mean that the individual will suffer from a disease because a number of different enzymes may compete to modify the same metabolic step. Unless a critical enzyme is disabled, disease will not arise. To recognize a distinct and well-defined reason of metabolic disorder, therefore, even now remains a challenge. While the field of metabolism related research continues to grow and expand, we have gained much knowledge and insight into the impact of gene mutation as a causal factor of metabolic disorders and potential new techniques to be employed in the future. These innovative insights will be an important review from which future research may continue to grow and expand.

## Figures and Tables

**Table 1:** Metabolic enzymes deficiency disorders and genetic mutations.

Enzymes	Deficiency disorders	Mutations	Protein/amino acid/nucleotide	Mutation types	Gene location	Other complications	References
Glucose-6-phosphatase	Von Gierke's disease	c.130C>T	p.P44S	Missense	Chromosome 17q21	Single episode of myositis	[9, 133]
		c.346A>G	p.M116V	Missense	17q21	T-cell lymphopenia, monocytosis, anemia, bilateral inguinal herniae, undescended testes, Dursun syndrome, and thymus hypoplasia	[9, 134]
		c.347 T>A	p.M116K	Missense	17q21	Learning difficulties and hypogonadotrophic hypogonadism, mild mitral and tricuspid insufficiency	[9, 133, 135]
		c.461 T>C	p.L154P	Missense	17q21	Hypercellular marrow, myeloid hyperplasia, no maturation arrest, frontal bossing, depressed nasal bridge, upturned nose, retrognathia, and learning difficulties	[136]
		c.554 T>C	p.L185P	Missense	17q21	Pulmonary valve stenosis	[137]
		c.623 T>G	p.L208R	Missense	17q21	Tricuspid insufficiency	[138]
		c.758G>A	p.R253H	Missense	17q21	Reduced mature neutrophils, discontinuous labia majora and minora, close set, down sloping eyes, low set ears, and bilateral cryptorchidism	[137–140]
		c.778G>C	p.G260R	Missense	17q21	Micropenis, mild developmental delay, hypoplastic nipples, malar flattening, reduced mature neutrophils, and cryptorchidism	[137, 139, 141]
		c.779G>A	p.G260D	Missense	17q21	Maturation arrest at myelocyte/promyelocyte stage, triangular face, frontal bossing, micrognathia, depressed nasal bridge, and cutis laxa	[139]
		c.144C>A	p.Y48X	Frame-shift and splice-site	17q21	Cryptorchidism, bilateral inguinal hernia, and cleft palate	[137]
		c.190_210del	p.T64_I70del	Frame-shift and splice-site	17q21	Granulomatous inflammatory bowel disease, splenomegaly, digital clubbing, and short stature	[138, 140]
		c.210delC	p.I70fsX46	Frame-shift and splice-site	17q21	Triangular face, depressed nasal bridge, growth retardation, enlarged anterior pituitary lobe, and maturation arrest at myelocyte/promyelocyte stage	[137, 142]
		c.218 + 1G >A	—	Frame-shift and splice-site	17q21	Reduced mature neutrophils and increased reticular staining, right inguinal hernia, bilateral cryptorchidism, and frontal bossing	[137]
		c.416G>T	—	Frame-shift and splice-site	17q21	Maturation arrest at myelocyte/promyelocyte stage, failure to thrive	[137]
		c.[766_777del]	p.[S255fs]	Frame-shift and splice-site	17q21	Broad face, prominent ears, small nose, big mouth, narrow forehead, short philtrum, and bilateral inner ear hearing loss	[137]
		c.131C>T; 758 G>A	p.P44L; R253H	Frame-shift and splice-site	17q21	Flat malar region, short philtrum, splenomegaly, and right ptosis	[139]
		c.210delC; 348G>A	p.I70fsX46; M116I	Frame-shift and splice-site	17q21	Triangular face, prominent upper lip, depressed tip of nose, and narrow thorax	[139, 142]
		c.677 + 1G>A; 829C>T	p.Gln277X	Frame-shift and splice-site	17q21	—	[9]
Fructose-1,6-bisphosphatase		581T>C,	F194S	—	9q22.2-q22.3	Hepatomegaly, acidosis, ketonuria, elevated uric acid level, and increased lactate and lipid level	[47]
		851C>G,	P284R	—	—	Vomiting, drowsiness, tachypnea, and hepatomegaly	[47]
		960/961insG	—	—	—	Glyceroluria	[45]
		A177D	530C-A	Missense	—	—	[45]
		E30X	88G-T	Nonsense	—	—	[45]
		V325A	974T→C	Nonsense	—	—	[45]
Ribose-phosphate isomerase		c.540delG	—	Frame-shift	2p11.2	—	[113, 143]
		c.182C>T,	p.A61V	Missense	2p11.2	—	[113, 143]
Transaldolase		c.512_514delCCT	p.Ser171del	Homozygous	11p15.5-p15.4 1p34.1-p33 (pseudo-gene)	Aortic coarctation, tubulopathy, splenomegaly, and neonatal oedema	[118]
		c. 575G>A	p.Arg192His	Missense	11p15.5-p15.4 1p34.1-p33 (pseudo-gene)	Glomerular proteinuria, large venous duct, cardiomyopathy, and splenomegaly	[117]
		c.512_514delCCT	p.Ser171del	—	11p15.5-p15.4 1p34.1-p33 (pseudo-gene)	Nephrocalcinosis	[116]
		c.574C>T	p.Arg192Cys	—	11p15.5-p15.4 1p34.1-p33 (pseudo-gene)	Tubulopathy	[118]
		c.575G>A	p.Arg192His	Missense	11p15.5-p15.4 1p34.1-p33 (pseudo-gene)	Neonatal oedema, liver fibrosis, hepatosplenomegaly, and anaemia	[115]
Succinate dehydrogenase		IVS1+1G4T	c.72+1G4T	—	SDHB 1p35-p36.1	Paraganglioma, pheochromocytoma	[102]
		IVS4+1G4C	c.423+1G4C, c.423+1G4A	—	SDHB 1p35-p36.1	Progressive external ophthalmoplegia	[102, 144]
		c.45_46insCC	—	—	SDHB 1p35-p36.1	Paraganglioma, pheochromocytoma, and progressive external ophthalmoplegia	[102]
		c.43C4T	—	—	SDHC 1q21	Optic atrophy, ataxia, progressive myopathy, and developmental delay	[102]
		IVS5+1G4A	c.405+1G4A	—	SDHC 1q21	Progressive external ophthalmoplegia	[102, 145]
		c.57delG	—	—	SDHD 11q23	Optic atrophy, ataxia, progressive myopathy, developmental delay, and progressive external ophthalmoplegia	[102]
Glucose-6-phosphate dehydrogenase		202(G→A)/ 376(A→G)	—	—	Xq28	Hemolytic anemia	[109, 110]
		563C→T	—	—	Xq28	Cyanosis, headache, fatigue, tachycardia, dyspnea, lethargy, lumbar/substernal pain, abdominal pain, splenomegaly, hemoglobinuria, and/or scleral icterus	[104, 109, 110]
		1003G→A	—	—	Xq28	Splenomegaly, hemoglobinuria, and/or scleral icterus	[104, 109, 110]
		1376G→C	—	—	Xq28	Cyanosis, headache, fatigue, tachycardia, lethargy, lumbar/substernal pain, and abdominal pain	[80, 104, 109, 110]
		68 Val→Met	—	—	Xq28	Hemoglobinuria	[146]
		126 Asn→Asp	—	—	Xq28	Lethargy, lumbar/substernal pain	[146]
Fumarase	Fumaric aciduria	c.1358T4C	p.L453P	—	1q42.1	Neurological impairment, microcephaly	[147]
		c.653T4C1	[p.L218P]	—	1q42.1	Encephalopathy, seizures, vomiting, and hypotonia	[96, 147]
		c.512G4A	p.S171N	—	1q42.1	Neurological impairment, encephalopathy, seizures, vomiting, and hypotonia	[96]
		A265T	—	Missense	1q42.1	Encephalopathy, seizures, vomiting, and hypotonia	[147]
		D383V	—	Missense	1q42.1	Microcephaly, seizures, developmental delay, or mental retardation	[147]
		F269C	—	Missense	1q42.1	Encephalopathy, mental retardation	[147]
		K187R	—	Missense	1q42.1	Microcephaly, seizures	[147]
		W458X	—	Nonsense	1q42.1	Seizures, vomiting, and hypotonia	[147]
Pyruvate dehydrogenase complex	Leigh disease	A1133G	—	Missense	Xp22 (E1*α*)	Hypotonia, developmental delay	[71, 148]
		C214T	—	Missense	Xp22 (E1*α*)	Corpus callosum abnormalities	[81]
		C615A	—	Missense	Xp22 (E1*α*)	Peripheral neuropathy	[81]
		R263G	—	Missense/nonsense	Xp22 (E1*α*)	Corpus callosum abnormalities, seizures, hypotonia, developmental delay, and peripheral neuropathy	[81, 149]
		R72	—	Missense/nonsense	Xp22 (E1*α*)	Callosal agenesis/dysgenesis, cerebral atrophy	[149]
		R378	—	Missense/nonsense	Xp22 (E1*α*)	Ataxia, relapsing dystonia	[149]
N-Acetylglutamate synthetase deficiency		TGG→TAG	Trp324Ter	Null mutation	17q21.31	Hyperammonemia	[126]
		1025delG	—	Deletion	17q21.31	Vomiting, altered consciousness, seizures, and coma	[126]
		C200R	c.598T>C	Missense	17q21.31	Chronic headaches, nausea	[130, 150]
		S410P	c.1228T>C	Missense	17q21.31	Hyperammonemia, altered level of consciousness, seizures, coma, and chronic headaches	[130, 150]
		A518T	c.1552G>A	Missense	17q21.31	Vomiting, altered level of consciousness, seizures, coma, and neurological impairment	[120, 130, 150]
		L430P	c.1289T>C	—	17q21.31	Hyperammonemia, vomiting, coma, chronic headaches, and nausea	[130, 150]
		W484R	c.1450T>C	—	17q21.31	Hyperammonemia, neurological impairment	[123, 130, 150]
